# Evaluation of the genotoxic potential of two rice herbicides, cyhalofop-butyl and quinclorac, in *Drosophila melanogaster*

**DOI:** 10.7717/peerj.21747

**Published:** 2026-10-05

**Authors:** Serap Kocaoğlu-Cenkci

**Affiliations:** Faculty of Health Sciences, Department of Nutrition and Dietetics, Akdeniz University, Antalya, Turkey

**Keywords:** *Drosophila* wing spot test, Genotoxicity, Herbicides in rice production

## Abstract

Cyhalofop-butyl, an aryloxyphenoxypropionate acetyl-CoA carboxylase (ACCase) inhibitor, and quinclorac, a quinolinecarboxylic acid herbicide with auxin-like activity, are commonly used in rice production. This study evaluated the genotoxic potential of the herbicides cyhalofop-butyl and quinclorac in the *in vivo Drosophila melanogaster* smart assay. Trans-heterozygous larvae obtained from crosses between the multiple wing hairs (*mwh*) and *flare*-3 (*flr*^3^) strains were exposed to four concentrations (1, 2, 5, and 10 ppm) of each herbicide. Both herbicides significantly increased clone frequencies compared with the negative control. Cyhalofop-butyl exhibited a concentration-related increase in total spot frequencies, whereas quinclorac consistently elevated clone frequencies across all tested concentrations without a clear dose-dependent pattern. To the best of our knowledge, this is the first report demonstrating the genotoxic potential of cyhalofop-butyl. These findings emphasize the need for careful use of herbicides in rice production and support further investigation of alternative products with lower or no genotoxic potential.

## Introduction

Rice (*Oryza sativa L*.) is among the most important staple food crops worldwide, and its production has steadily increased in response to the growing global population and rising demand. Although China, India, Indonesia, Bangladesh, and Vietnam are the leading producers, rice cultivation is practiced worldwide (Kaya et al., 2017).

Keeping rice fields flooded provides favorable conditions for the growth and spread of weeds (Kraehmer et al., 2016). Weeds compete with rice plants for light, space, and nutrients, leading to reduced yield and grain quality (Peng, Tang & Zou, 2009; Jiang et al., 2024). Therefore, effective weed management is crucial for sustainable and high-yielding rice production. Although manual weed control is feasible in small-scale production, the use of herbicides has become a widespread weed management strategy because manual control is impractical in large-scale agriculture.

Cyhalofop-butyl is a herbicide belonging to the aryloxyphenoxypropionate group; it inhibits the acetyl-CoA carboxylase (ACCase) enzyme, thereby blocking fatty acid biosynthesis and causing growth arrest and death in susceptible weed species such as *Echinochloa crus-galli*. Quinclorac, on the other hand, is a quinoline carboxylic acid derivative herbicide that exhibits auxin-like activity, leading to uncontrolled cell elongation, ethylene synthesis, and ultimately plant death (Cengiz & Cetin, 2025).

The careless, improper, or excessive use of herbicides can cause direct or indirect toxic effects on non-target organisms in rice paddy ecosystems (Solomon et al., 2014; Mengüç, 2018). In these ecosystems, not only microorganisms such as microalgae and cyanobacteria but also higher organisms such as amphibians, crabs, and fish are vulnerable to herbicide exposure (Abdullah et al., 1997; Liu et al., 2011). Furthermore, in some rice-growing areas, the production of various agricultural crops can be carried out alongside fish farming (Timor, 2002), and drainage water can be reused for vegetable irrigation (Kaya et al., 2023). The detection of quinclorac residues in the leaves and flowers of tomato plants and reports of its potential to be transferred from flowers to fruits (Lovelace et al., 2009), as well as its detection in food sources such as rice products (Jiménez-López, Llorent-Martínez & Ruiz-Medina, 2020) and honey (Thompson et al., 2023), indicate this compound’s persistence in agricultural systems and its potential for transfer through the food chain.

Field observations (Nagafuchi, Inoue & Ebise, 1994; Ebise & Inoue, 2002; Watanabe, Kakegawa & Vu, 2006; Watanabe et al., 2007) and modeling studies (Nagafuchi, Inoue & Ebise, 1994; Nguyen et al., 2001; Watanabe, Takagi & Vu, 2006) have shown that herbicides are transported to a significant extent *via* surface runoff, particularly during the early period after application when concentrations are highest, as a result of excessive irrigation, heavy rainfall, or controlled drainage carried out by farmers. Field monitoring studies in rivers supplying drinking and domestic water to cities (Kondo et al., 2012; Kubo et al., 2012; Sudo et al., 2012; Matamoros et al., 2020; Calvo et al., 2021; Syafrudin et al., 2021; Tan et al., 2021) and lakes (Sudo et al., 2004; Kawakami et al., 2005; Ishihara, 2008) have shown that herbicides, other pesticides, and their degradation or transformation products are frequently detected. Furthermore, herbicides can be transported from multiple agricultural areas *via* river flows and reach distant regions and non-target organisms. This situation highlights the importance of evaluating the safety and genotoxic potential of widely used herbicides—particularly cyhalofop-butyl and quinclorac—to protect non-target organisms and ensure sustainable rice production.

Accordingly, the present study investigated the genotoxic potential of cyhalofop-butyl and quinclorac—two compounds widely used in rice cultivation that can reach humans and non-target organisms through environmental pathways. For the genotoxicity assessment, the eukaryotic model organism *Drosophila melanogaster*, which is widely used in genetic, toxicological, and biomedical research, was selected (Bernards & Hariharan, 2001; Link & Bellen, 2020). *D. melanogaster* is widely used in various genotoxicity studies to evaluate the effects of physical, biological, and chemical agents on genetic material (Kaya, Kocaoğlu & Demir, 2006; Demir, Kaya & Kocaoglu Cenkci, 2013). In this context, the genotoxic potential of cyhalofop-butyl and quinclorac was evaluated using an *in vivo* wing somatic mutation and recombination test (SMART) performed on trans-heterozygous *D. melanogaster* individuals.

## Materials & Methods

### Tested herbicides

In this study, commercial formulations of cyhalofop-butyl (20% EC, emulsifiable concentrate) and quinclorac (25% OD, oil dispersion) were used to evaluate their genotoxic potential. Ethyl methanesulfonate (EMS; CAS No. 62-50-0; Sigma-Aldrich) at a concentration of 1 mM was used as a positive control (Marcos & Carmona, 2013), whereas distilled water served as the negative control. Based on previous studies (Concenço et al., 2009; Xia et al., 2018; Güner, 2020; Cheng et al., 2021; Duan et al., 2022; Ou-Yang et al., 2023; Xiong et al., 2023; Ferrer et al., 2024), four concentrations (1, 2, 5, and 10 ppm) were selected for both herbicides. The main properties of the tested herbicides are presented in Fig. 1.

**Figure 1:**
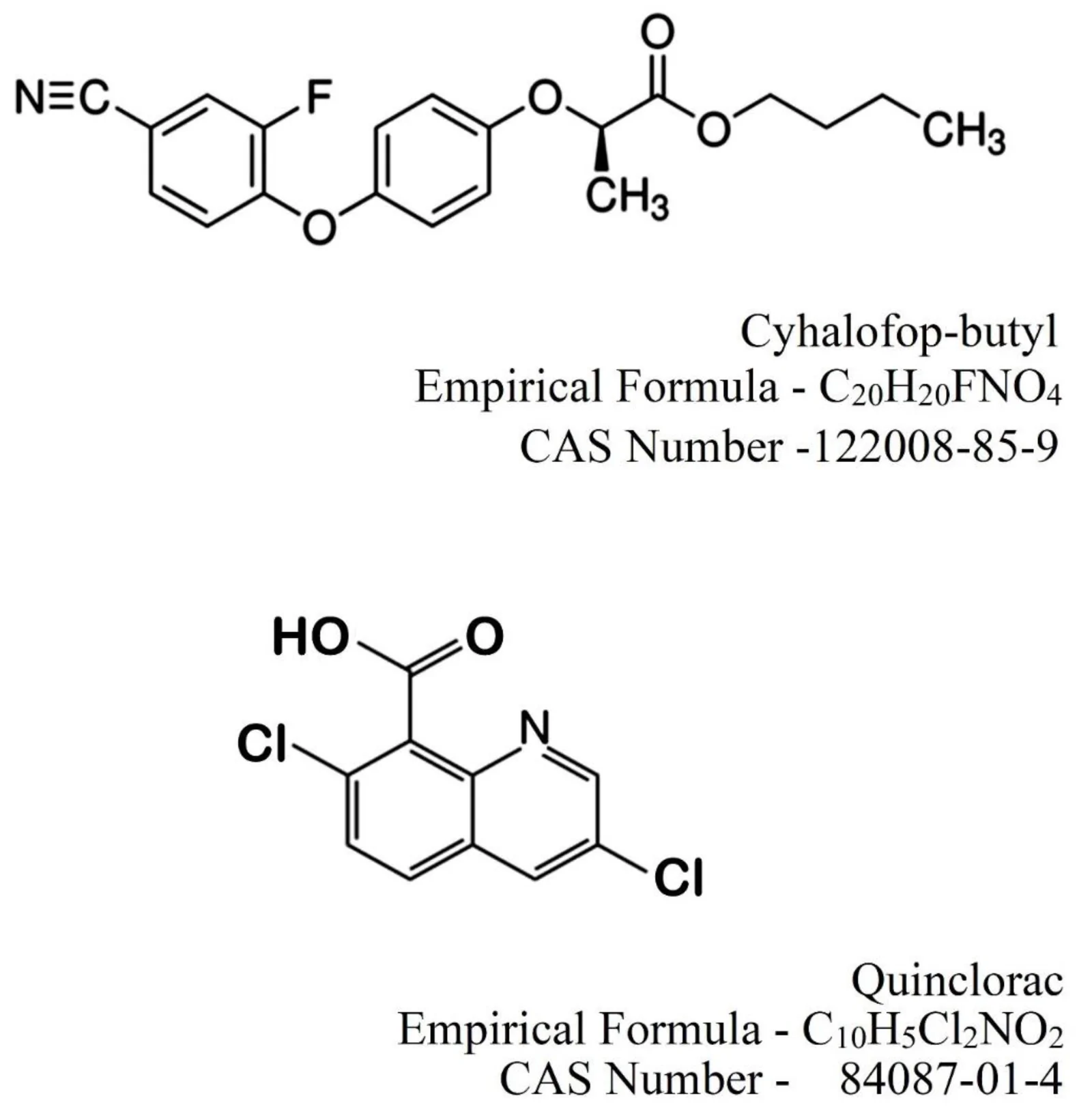
Chemical properties of the tested herbicides.

### Drosophila strains

Two *Drosophila melanogaster* strains were used in this study: the multiple wing hair strain (genotype *mwh/mwh*) and the *flare*-3 strain (genotype *flr*^3^/*In(3LR)TM3, BdS*). Detailed information on the genetic symbols and their descriptions is available in Lindsley & Zimm (1992).

### Experimental procedure

The wing spot (SMART) test is based on the detection of loss of heterozygosity in the somatic cells of *Drosophila melanogaster* larvae (Graf et al., 1984). Trans-heterozygous larvae were obtained by crossing *flr*^3^ virgin females with *mwh* males. Virgin females and males aged 3–7 days were used for crossing. To ensure fertilization and embryogenesis, male and female flies were kept together in the same culture medium for at least 24 h. Subsequently, the flies were transferred to fresh Lewis medium (Lewis & Bacher, 1968) and allowed to oviposit for 8 h. This procedure enabled the collection of trans-heterozygous larvae at the same developmental stage.

For the treatment medium, 4.5 g of ready-to-use *Drosophila* Instant Medium (Carolina Biological Supply Company, Burlington, NC, USA) was placed into plastic vials (25 mm × 95 mm, diameter × height). The food was rehydrated with 9 mL of freshly prepared test chemical solutions immediately before treatment. Larvae hatched from the collected eggs were separated from the culture medium when they reached 72 ± 4 h of age (third instar larvae) by gently washing the culture medium with tap water. The larvae were collected using a fine-mesh sieve and rinsed with tap water to remove residual medium. Approximately 100 larvae were transferred into each treatment vial. Three replicate treatment vials were prepared for each concentration. The larvae were maintained in an incubator until adult flies emerged. All experiments were conducted under controlled conditions at 25 ± 1 °C and approximately 65% relative humidity.

### Preparation and microscopic analysis of the wings

A single independent experiment was performed, with three replicate exposure vials for each treatment concentration. Following metamorphosis, trans-heterozygous flies were collected and preserved in 70% ethanol at 4 °C. At the end of the exposure period, flies from the three replicate vials were pooled and treated as a single sample for subsequent analyses. The wings were dissected under a Nikon SMZ645 stereomicroscope (Nikon, Tokyo, Japan) and mounted on microscope slides using Faure’s solution (30 g gum arabic, 30 mL glycerol, 50 g chloral hydrate, and 50 mL distilled water). Only trans-heterozygous wings (*mwh/flr*^3^) were analyzed, as the study focused on genotoxic effects rather than the detection of mutational mechanisms. Wing preparations were prepared from 40 individuals (80 wings) randomly selected from normal-winged flies for each concentration. Frei & Wurgler (1995) noted that 80 wings per concentration are sufficient for statistical analysis. The prepared specimens were examined using a Nikon YS100 light microscope (Nikon, Tokyo, Japan; 400× magnification) to assess the presence of cell clones exhibiting morphological abnormalities in the wing feathers. The wings were evaluated for (1) small (1–2 cells) single spots, (2) large (>2 cells) single spots (both with the *mwh* or *flr*^3^ phenotype), and (3) twin spots (adjacent clones with *mwh* and *flr*^3^ phenotypes) (Graf et al., 1984).

### Statistical analysis

Statistical evaluation was performed according to the multiple decision procedure described by Frei & Wurgler (1988), which classifies responses as positive, weakly positive, negative, or inconclusive. The conditional binomial test described by Kastenbaum & Bowman (1970) was applied at a 5% significance level.

## Results

The genotoxic potential of the herbicides cyhalofop-butyl and quinclorac, commonly applied for weed control in paddy production, was evaluated using the *Drosophila melanogaster* wing SMART. EMS (one mM), an alkylating agent with well-established genotoxic activity, was included as a positive control to confirm the validity of the experimental system. The results are summarized in Table 1. Four concentrations (1, 2, 5, and 10 ppm) of each herbicide were tested and compared with the negative control (distilled water) using statistical analyses.

**Table 1:** Frequency of spot clones in the *Drosophila* wing SMART test following larval treatment with cyhalofop-butyl and quinclorac.

Concentrations	No. of wings (*N*)	Small single spots (1–2 cells) (*m* = 2)			Large single spots (>2 cells) (*m* = 5)			Twin spots (*m* = 5)			Total *mwh* spots^a^ (*m* = 2)			Total spots (*m* = 2)			Frequency of clone formation per 10^5^ cells^b^
		No	Fr	D	No	Fr	D	No	Fr	D	No	Fr	D	No	Fr	D	
Distilled water	80	12	0.15		2	0.03		1	0.01		15	0.19		15	0.19		0.77
1 mM EMS	80	142	1.78	+	114	1.43	+	36	0.45	+	235	2.94	+	292	3.65	+	14.96
**Cyhalofob-butyl**																	
1 ppm	80	24	0.30	+	1	0.01	+	0	0.00	+	25	0.31	+	25	0.31	+	1.28
2 ppm	80	24	0.30	+	2	0.03	+	1	0.01	+	27	0.34	+	27	0.34	+	1.38
5 ppm	80	25	0.31	+	4	0.05	+	2	0.03	+	31	0.39	+	31	0.39	+	1.59
10 ppm	80	37	0.46	+	3	0.04	+	0	0.00	+	40	0.50	+	40	0.50	+	2.05
**Quinclorac**																	
1 ppm	80	23	0.29	+	1	0.01	+	4	0.05	+	28	0.35	+	28	0.35	+	1.43
2 ppm	80	23	0.29	+	4	0.05	+	2	0.03	+	29	0.36	+	29	0.36	+	1.49
5 ppm	80	24	0.30	+	3	0.04	+	2	0.03	+	29	0.36	+	29	0.36	+	1.49
10 ppm	80	28	0.35	+	2	0.03	+	2	0.03	+	32	0.40	+	32	0.40	+	1.64

**Notes.**

No number Fr frequency D statistical diagnosis according to Frei & Wurgler (1988) + positive − negative *i* inconclusive *m* multiplication factor, probability levels *α* = *β* = 0.05

a Considering *mwh* clones from single and twin spots.

b Frequency of clone formation: *mwh* clones/wings/24,400 cells (without size correction) (Graf and Van Schaik, 1992).

Spots were scored and classified as: (1) small (1–2 cells) single spots, (2) large (>2 cells) single spots, both with *mwh* or *flr*^3^ phenotype, and (3) twin spots (phenotypes *mwh* and *flr* in adjacent clone).

Analysis of the data obtained for cyhalofop-butyl revealed no significant increases in the frequencies of large single and twin spots at any tested concentration; comparisons with the distilled water control indicated that these responses were inconclusive. In contrast, significant increases were observed in the frequencies of small single spots, total *mwh* spots, and total spots (Table 1). Furthermore, total spot frequencies showed a dose-dependent increase, with the highest frequency observed at 10 ppm (Fig. 2). Based on the significant increases in small single spots, total *mwh* spots, and total spot frequencies, cyhalofop-butyl treatment was classified as positive in the SMART assay under the tested experimental conditions.

**Figure 2:**
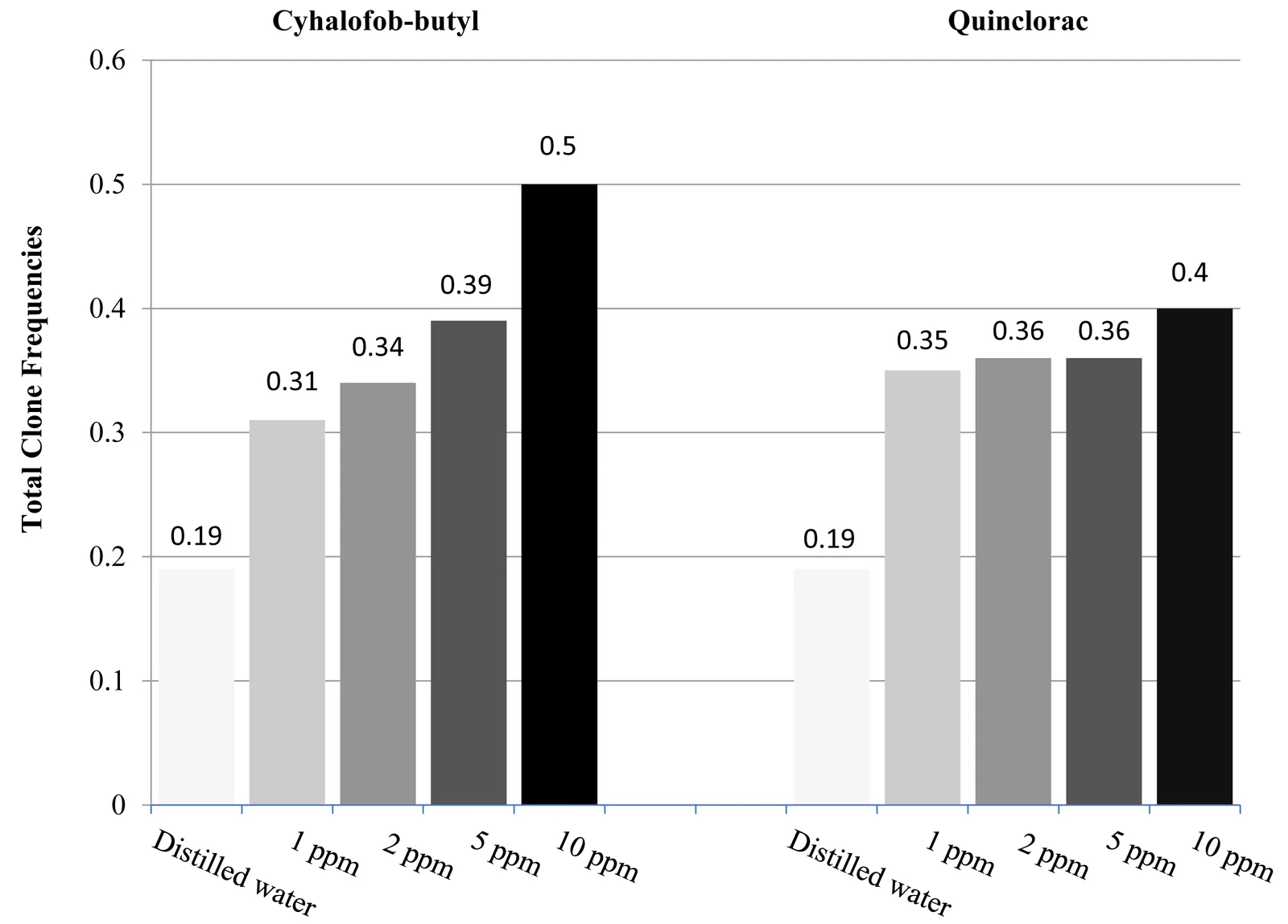
Total clone frequency distributions in *mwh*/*flr*^3^ individuals exposed to cyhalofop-butyl and quinclorac.

Assessment of the data for quinclorac revealed no significant increases in the frequencies of large single and twin spots; comparisons with the distilled water control indicated that these responses were inconclusive. In contrast, significant increases were observed in small single spots, total *mwh* spots, and total spot frequencies compared with the control group (Table 1). Total spot frequencies remained higher than control values at all tested concentrations, although only minor differences were observed among doses (Fig. 2). Based on the increases in small single spots, total *mwh* spots, and total spot frequencies, quinclorac treatment was classified as positive in the SMART assay under the tested experimental conditions.

## Discussion

Quinclorac and cyhalofop-butyl are among the herbicides widely used worldwide to increase rice yields. Because rice is cultivated under flooded conditions, these herbicides can be transported into surrounding aquatic environments, leading to unintended exposure of non-target organisms. Previous studies evaluating the toxicological effects of quinclorac and cyhalofop-butyl have primarily focused on aquatic organisms and have shown that oxidative stress is one of the main mechanisms underlying their toxicity. However, information regarding the genotoxic potential of these herbicides remains limited, particularly in *in vivo* eukaryotic systems. The findings of the present study, obtained using the *Drosophila* wing SMART assay, are discussed in comparison with available literature, with particular emphasis on the potential genotoxic effects of quinclorac and cyhalofop-butyl.

Previous investigations of quinclorac and cyhalofop-butyl toxicity have been conducted using various biological models, providing important insights into their toxicological mechanisms and environmental relevance. Among these models, aquatic organisms, particularly fish species, have been frequently used to evaluate the adverse effects of these herbicides and have contributed substantially to understanding their oxidative stress-related toxicity (Song et al., 2022). Quinclorac has been reported to induce oxidative damage in the liver of silver catfish (*Rhamdia quelen*), resulting in increased lipid peroxidation and impairment of antioxidant enzymatic defense systems (De Menezes et al., 2014). Similar findings have been reported in the gills of juvenile *R. quelen* (Persch et al., 2017) and in common carp (*Cyprinus carpio*), where quinclorac exposure caused alterations in biochemical parameters, depletion of non-enzymatic antioxidants, and pronounced lipid peroxidation in several tissues (De Menezes et al., 2012). Furthermore, hepatotoxic and cytotoxic effects mediated by oxidative stress have been demonstrated in zebrafish liver cell lines (Ferrer et al., 2024). Additional studies have shown that quinclorac exposure induces hematological alterations in Nile tilapia (*Oreochromis niloticus*) (Fathy et al., 2019) and increases lipid peroxidation in bullfrog (*Lithobates catesbeianus*) tadpoles (Dornelles & Oliveira, 2016).

Collectively, these findings indicate that quinclorac induces oxidative damage across multiple aquatic organisms, suggesting a central role for oxidative stress in its toxic mode of action.

Despite the growing body of evidence regarding quinclorac-induced toxicity, information concerning its genotoxic potential remains limited. Xiping (2006) demonstrated a dose-dependent increase in deoxyribonucleic acid (DNA) damage in erythroblast cells using the Comet assay. In the same study, the Ames test conducted with TA97, TA98, and TA100 bacterial strains revealed increased numbers of revertant colonies both in the presence and absence of metabolic activation, suggesting mutagenic activity. Similarly, Da Conceição & Protti (2012) reported a significant increase in micronucleus frequency in embryonic erythrocytes of hens (*Gallus gallus domesticus*) exposed to quinclorac. These findings provide direct evidence of the genotoxic potential of quinclorac across different biological systems and suggest that oxidative stress-mediated cellular damage may contribute to its genotoxic effects.

The results of the present study are consistent with these previous reports. All tested concentrations of quinclorac (1, 2, 5, and 10 ppm) produced statistically significant increases in clone frequencies in the SMART assay, indicating mutagenic and/or recombinogenic activity. Total spot frequencies remained above control values at all tested concentrations, although only minor variations were observed between doses. These findings corroborate earlier reports demonstrating quinclorac-induced DNA damage (Xiping, 2006) and extend the current body of evidence by showing that quinclorac can induce somatic genetic alterations *in vivo* in *Drosophila melanogaster*.

Compared with quinclorac, information regarding cyhalofop-butyl toxicity and genotoxicity remains more limited. Nevertheless, recent studies have shown that cyhalofop-butyl induces hepatotoxicity and immunotoxicity in zebrafish embryos (Xiong et al., 2023) and promotes immune inflammation along the gut–liver axis in adult zebrafish through disturbances in glucose and lipid metabolism (Duan et al., 2025). In addition, cyhalofop-butyl exposure has been associated with concentration-dependent oxidative stress and apoptosis, characterized by dysregulation of apoptosis-related and anti-apoptotic genes (Cheng et al., 2021). Developmental toxicity, including lipid accumulation, abnormal embryonic development, and impaired cardiac morphology and function, has also been consistently reported (Cheng et al., 2021; Peng et al., 2025).Furthermore, alterations in reproductive hormone levels and reduced fertility have been observed following exposure to cyhalofop-butyl (Duan et al., 2022). Zhu et al. (2015) also reported concentration-dependent teratogenic effects and significant alterations in the expression of genes encoding antioxidant proteins and apoptosis-related factors, highlighting oxidative stress and apoptosis as key mechanisms underlying cyhalofop-butyl-induced cardiotoxicity.

Although available studies clearly demonstrate the toxicological effects of cyhalofop-butyl, direct evidence regarding its genotoxicity has been lacking. In the present study, all tested concentrations of cyhalofop-butyl (1, 2, 5, and 10 ppm) significantly increased clone frequencies in the SMART assay, indicating mutagenic and/or recombinogenic activity. Moreover, total spot frequencies exhibited a dose-dependent increase, with the highest frequency observed at 10 ppm. To the best of our knowledge, this is the first report demonstrating the genotoxic potential of cyhalofop-butyl in an *in vivo* eukaryotic test system. Considering that oxidative stress is a well-established mechanism leading to DNA strand breaks, base modifications, and chromosomal damage, the genotoxic effects observed in this study may be associated with oxidative stress pathways previously reported for this compound. However, further studies using complementary genotoxicity assays are required to elucidate the underlying mechanisms in more detail. Taken together with previous reports of oxidative stress induced by cyhalofop-butyl and quinclorac, oxidative damage may represent a common mechanistic link underlying the genotoxic effects observed for both herbicides.

The oxidative stress responses reported in previous studies following quinclorac and cyhalofop-butyl exposure are consistent with the increased clone frequencies observed in the present study. These findings suggest that oxidative DNA damage may contribute to the genotoxic potential of these herbicides. A comparison of the two herbicides revealed similar patterns of genotoxic responses at the tested concentrations. Nevertheless, cyhalofop-butyl exhibited numerically higher clone frequencies than quinclorac at the highest tested concentration, suggesting a stronger response under the experimental conditions of the present study. Considering the widespread agricultural application of both compounds and the limited knowledge regarding their genetic safety, these findings emphasize the need for further mechanistic investigations and long-term genotoxicity assessments.

However, it should be considered that the genotoxic potential of quinclorac and cyhalofop-butyl in the present study was evaluated using only normal-winged descendants within the SMART assay. Therefore, the increases observed in clone frequencies reflect the overall genotoxic effect but do not allow the relative contributions of mutational and recombinational events to this effect to be distinguished. Future studies using different SMART cross designs, including high bioactivation strains exhibiting elevated cytochrome P450 activity, may provide more comprehensive insights into the mechanisms underlying the observed genotoxic effects.

## Conclusions

This study demonstrated that cyhalofop-butyl and quinclorac exhibit genotoxic potential in *Drosophila melanogaster*, as evidenced by statistically significant increases in clone frequencies in the wing SMART assay. These findings provide the first evidence of the genotoxic potential of cyhalofop-butyl and contribute to the limited information available on quinclorac-induced genotoxicity. Considering the widespread use of these herbicides in rice production, the results highlight the importance of further risk assessments and monitoring programs. Additional studies employing different model organisms and molecular approaches are needed to better elucidate their potential impacts on environmental and human health.

##  Supplemental Information

10.7717/peerj.21747/supp-1 Data S1 Frequency of mutant clones in the *Drosophila* wing SMART test following larval treatment with cyhalofop-butyl and quinclorac Each data point indicates frequency of mutant clones in the *Drosophila* wing SMART test following larval treatment with cyhalofop-butyl and quinclorac

## Additional Information and Declarations

### Data Availability

The following information was supplied regarding data availability: The raw data is available in the Supplementary Files.
